# Effects of forage level and chromium-methionine chelate supplementation on performance, carcass characteristics and blood metabolites in Korean native (Hanwoo) steers

**DOI:** 10.1186/s40781-015-0043-7

**Published:** 2015-03-21

**Authors:** Kyung-Il Sung, Jalil Ghassemi Nejad, Seok-Man Hong, Sang-Jip Ohh, Bae-Hun Lee, Jing-Lun Peng, Do-Hyeon Ji, Byong-Wan Kim

**Affiliations:** College of Animal Life Sciences, Kangwon National University, Chuncheon, 200-701 Republic of Korea

**Keywords:** Blood metabolites, Carcass characteristics, Chromium methionine, Forage level, Hanwoo steers

## Abstract

A feeding trial was carried out to determine the effects of chromium methionine (Cr-Met) chelate and forage level over two years, 1^st^ fattening and 2^nd^ fattening period on growth parameters, carcass characteristics and blood metabolites of 46 Korean native (Hanwoo, Bos Taurus, BW = 183 ± 44 kg) steers. Treatments were: 1) Steers in the low forage (LF) group were fed diets that consisted of 60% concentrate and 40% forage; 2) Steers in the high forage (HF) group were fed diets that consisted of 40% concentrate and 60% forage. Following the 1^st^ fattening period, steers (BW = 480 ± 37.6 kg) were randomly assigned to four treatment groups: LF (40 F plus no Cr-Met supplementation in the 2^nd^ fattening period), LFCM (40LF plus added 400 ppb of Cr-Met during the 2^nd^ fattening period), HF (60 F plus no added Cr-Met during the 2^nd^ fattening period) and HFCM (60 F plus added 400 ppb of Cr-Met in the 2^nd^ fattening period). Dry matter intake of the treatment diets did not differ during the raising and 1^th^ fattening period (P > 0.05). The ADG in the raising period showed no difference between the 40 F and 60 F groups (P > 0.05). Carcass characteristics including rib-eye area and meat yield index were higher in HF than the other treatment groups (P < 0.05). The HF group tended to show a higher (P = 0.08) marbling score than the LF group whereas the HFCM group showed a higher marbling score than the LFCM group (P < 0.05). HDL was higher and LDL lower in groups fed with Cr-Met than in other groups whereas glucose showed the lowest value in HF group (P < 0.05). Triglyceride (TG), Cholesterol, PUN and total protein (TP) were the same among all treatment groups (P > 0.05). The Insulin concentration in the blood was significantly higher for the HFCM group than for the LF, LFCM and HF groups (P < 0.05). It is concluded that supplementation of chromium-methionine chelate could improve meat quality in beef steers.

## Background

Chromium is known to be partially liable in blood cholesterol regulation. Chromium-Met chelate absorbs directly through the intestinal cell membrane in an intact form [1]. Therefore, availability of Cr-Met chelate remains high [2]. Chromium is an essential trace mineral that has a principal role in glucose, protein and fat metabolisms in animal tissues [3]. Chromium supplementation of diets has been demonstrated to improve performance and decrease stress conditions in cattle. Cr supplementation has also been employed to manipulate the quality of meat due to its biological function on body fat and muscle metabolism [4], however, factors such as the level of Cr supplementation, nutrients, chromium levels in basal diet, breed and species may intervene with these functions. Therefore, a dietary Cr-Met chelate supplementation was suggested as a new approach for production of low-fat and low-cholesterol meat from meat producing animals [3,5]. Wang et al. [6] reported that chromium supplementation impacts blood metabolites and improves carcass quality in swine. Moreover, chromium increases glucose tolerance by boosting insulin function, resulting in decrease of postprandial sugar level in blood [7]. In addition, Organic forms of chromium, such as yeast culture containing high Cr concentration, are usually more absorbable than inorganic forms [8]. Other organic forms of chromium, such as chromium propionate or chromium-methionine, have shown consistent positive effects on glucose metabolism, feed intake, and milk production in dairy cattle compared with other chromium compounds [9-11].

Furthermore, forage level in diets is influential for meat production and meat quality throughout the growing and fattening of beef steers [12,13]. Optimal level of forage in diets is dependent on mineral supplementation strategy. In high fiber diets, feeding grain as an energy source can suppress forage digestion [12].

Song et al. [3] reported that supplementation of Cr-Met Chelate improved carcass characteristics and performance of Holstein steers. However, the impact of forage level and Cr-Met supplementation over the periods (growing, 1^th^ fattening and 2^nd^ fattening period) on Hanwoo steers production has not been investigated. Therefore, the aim of this study was to evaluate the carcass characteristics and growth parameters of Hanwoo steers fed with Cr-Met chelate supplement in two stages. This continuous study aimed to investigate interaction between fiber levels in early feeding period and Cr-Met chelate supplementation in late fattening period.

## Methods

### Experimental period, animals, and treatments assignation

The experiment took place over 13 months and was located in Gangwon province in South Korea. The animal body weight was 183 ± 44 kg and 46 Korean native steers were used in this experiment. The experiment was conducted to inspect the effect of high and low levels of forage in diet on growth performance during the raising and 1^st^ fattening period (14-22 month age for Hanwoo). Treatments were: 1) Steers in the low forage (LF) group were fed diets that consisted of 60% concentrate and 40% forage (40 F) during the raising period and then *ad libitum* feeding of concentrate on the 1^st^ fattening period; 2) Steers in high forage (HF) group were fed 40% concentrate and 60% forage (60 F) diets during the raising period and then *ad libitum* feeding of concentrate on the 1^st^ fattening period. Throughout the raising and 1^st^ fattening period steers were randomly allotted to one of two dietary treatments based on birth date and body weight.

The experiment was continued by investigating the effects of forage level and Cr-Met on carcass characteristics and blood metabolites in the 2^nd^ fattening period (23-30 month age for Hanwoo) of Korean beef steers. Upon completion of the 1^st^ fattening period, the steers with average body weights of 480 ± 37.6 kg were randomly assigned to four treatment groups: LF (40 F plus no added Cr-Met in the 2^nd^ fattening period), LFCM (40 F plus added 400 ppb of Cr-Met in the 2^nd^ fattening period), HF (60 F plus no added Cr-Met during the 2^nd^ fattening period) and HFCM (60 F plus added 400 ppb of Cr-Met in the 2^nd^ fattening period).

### Experimental procedure

Forty-six Korean native steers with an average initial weight of 183 ± 44 kg were used in this trial. Table 1 demonstrates the ingredients and chemical composition of the experimental feed. Feed in the raising period in 40 F included concentrate and rice straw *ad libitum* with 1 kg alfalfa (as-fed). In the 60 F concentrate 2 to 5 kg (as-fed) and bermudagrass hay *ad libitum*. Then, in the 1^st^ fattening period (14-22 months for Hanwoo) in 40 F and 60 F both concentrate and rice straw *ad libitum*. The experimental design in the 2^nd^ fattening period (23-30 months for Hanwoo) was done in two levels of forage with and without Cr-Met. 40 F in the raising and 1^st^ fattening period + concentrate without (LF) and with (LFCM) Cr-Met. Also 60 F in the raising and 1^st^ fattening period + concentrate without (HF) and with (HFCM) Cr-Met. The experimental procedure and methods were approved by the animal welfare and ethics authority of Kangwon National University, Chuncheon, Korea.

**Table 1:** **Mean feed value of feed ingredients in the experimental diet**

		**DM**	**OM**	**CP**	**NDF**	**ADF**
		**%**	**% of DM**			
	^1)^	88.2	93.1	19.3	36.1	16.1
Concentrate	^2)^	89.5	92.2	16.1	42.7	13.6
	^3)^	89.9	93.2	14.1	26.7	8.1
Alfalfa hay		89.0	89.5	17.4	37.7	27.8
Bermudagrass hay		88.7	93.4	8.3	76.8	62.3
Rice straw		89.7	89.4	4.8	79.2	54.5

^1)^Raising period.

^2)^1^st^ fattening period.

^3)^2^nd^ fattening period.

### Measurements and sampling

Feed was collected once per month, and disclosures of the common feed ingredients were analyzed according to the methods of AOAC [14]. Neutral detergent fiber (NDF) and acid detergent fiber (ADF) contents were analyzed using Goering and Van Soest [15].

Each day at 09:00 am feed intake was measured by subtracting the amount of residue and was calculated by dividing two numbers. Body weight was measured every 60 days from the beginning of the experiment and a total of 7 measurements were calculated by dividing the days (initial body weight, I to VI). Average daily weight gain was measured subsequently.

Upon the completion of the feedlot experiment, the steers were slaughtered. Post-fasting weights and carcass weight, after a 24 hour chilling period in a cold room (2°C) were recorded followed by determination of back fat thickness (mm), rib-eye area (cm^2^), meat yield index, quantity grade, marbling score, meat color, fat color, texture, maturity, and quantity grade. Carcass characteristics were measured based on MIFAFF [16].

Blood samples were collected from the jugular by venipunture containing anticoagulant (Sodium heparin) for plasma separation. Collected blood was then centrifuged at 2500 × g for 15 minutes and the samples were stored at −20°C until tested. Plasma samples were later analyzed by using the automatic blood analyzer (Express Plus, Ciba-Coming, CA, USA) to measure the concentrations of triglyceride, cholesterol, HDL, LDL, glucose, PUN, total Protein, albumin, creatinine, calcium, and insulin.

### Statistics

A completely randomized design was used in this experiment. For the 2^nd^ fattening period, beef steers were allotted to treatment groups based on completely randomized design with 2 × 2 factorial arrangement. In this study main effects were significant and no interaction between level of forage and Cr-Methionine chelate was found. Therefore, interaction data was not shown in Tables. Statistical analysis for all parameters (growth and carcass characteristics) was conducted using the GLM procedure of SAS [17]. Significant differences were tested by Duncan’s multiple tests [18], carried out at a level of 5%, and differences among means with 0.05 < P < 0.10 was accepted as representing tendencies to differences.

## Results

### Performance in raising and 1^st^ fattening period

The average daily gain (ADG) in 40 F was lower (P < 0.05) than 60 F at the beginning of the raising period (Table 2). The same trend, however, was observed during the 1^st^ fattening period where ADG was significantly higher for the 60 F than the 40 F (P < 0.05).

**Table 2:** **Feed intake and body weight gain in Hanwoo steers fed with different levels of forage in raising and 1** ^**st**^ **fattening period**

**Items**	**Treatments**	**Sampling period**						
		**I**	**II**	**III**	**IV**	**V**	**VI** ^**4)**^	**Mean**
ADG^1)^ (kg/d)	LF	0.55^b^	0.85^a^	0.72^a^	0.76^a^	0.70^a^	0.31^b^	0.65^b^
	HF	0.83^a^	0.83^a^	0.74^a^	0.74^a^	0.73^a^	0.59^a^	0.74^a^
DMI^2)^		5.56	3.56	4.56	5.60	6.46	5.09	5.14
	LF	2.69	2.74	2.24	1.79	1.79	1.16	2.06
	HF	2.47	2.74	3.21	4.33	6.27	6.32	4.22
		3.53	3.93	4.58	4.28	1.79	1.08	3.22
FCR^3)^	LF	15.0^a^	7.4^a^	9.4^a^	9.72^b^	11.8^a^	20.2^a^	11.0^a^
	HF	7.2^b^	8.0^a^	10.5^a^	11.6^a^	11.0^a^	12.5^b^	10.0^b^

^1)^Average daily gain.

^2)^Dry matter intake.

^3)^Feed conversion ratio.

^4)^I-VI: Number of measurement.

^a,b^Significant differences occurred at (P < 0.05).

Total dry matter intake (DMI), concentrate + forage, showed no differences between the 40 F and 60 F groups overall seen in Table 2 (P > 0.05).

The feed conversion ratio in the raising period (Table 2) tended to be higher for the 40 F than 60 F; but during the 1^st^ fattening period there was significantly higher increase for the 60 F group than the 40 F group (P < 0.05).

### Carcass characteristics and 2^nd^ fattening period

Post-fasting weight, carcass weight and back fat thickness were not affected by treatment groups (P > 0.05).

Results of the 2^nd^ fattening period documented above B grade in both HF and HFCM groups in quantity grade (Table 3). The quality grade (Table 3) was shown to be above 72% in the first grade and it was shown to be higher in HFCM than the LF, LFCM and HF. The rib-eye area in HF group was higher (P < 0.05) than LF and LFCM groups whereas no difference was observed between HFCM group and other treatment groups. The result of the meat yield index showed a similar trend for the result of rib-eye area among the treatment groups.

**Table 3:** **Effect of forage level and Cr-Met chelate supplementation diets on carcass weight and meat quality obtained from native Korean beef steers (Hanwoo, Bos taurus)**

**Items**	**Treatment**			
	**LF** ^**1)**^	**LFCM** ^**2)**^	**HF** ^**3)**^	**HFCM** ^**4)**^
Post-fasting weight (kg)	578.33 ± 40.70^a^	579.00 ± 41.41^a^	571.67 ± 19.41^a^	570.71 ± 48.91^a^
Carcass weight (kg)	343.67 ± 26.59^a^	344.25 ± 29.36^a^	345.33 ± 11.33^a^	339.86 ± 31.67^a^
Meat quantity				
Back fat thickness (mm)	10.67 ± 4.80^a^	10.55 ± 4.58^a^	7.33 ± 2.07^a^	10.43 ± 2.87^ab^
Rib-eye area (cm^2^)	75.67 ± 7.17^b^	76.15 ± 6.33^b^	84.67 ± 6.65^a^	68.10 ± 1.12^ab^
Meat yield index	57.56 ± 2.26^b^	67.64 ± 2.08^b^	69.65 ± 1.24^a^	68.10 ± 1.12^ab^
Quantity grade (A:B:C) (%)	33:50:17	25:50:25	50:50:00	29:71:0
Meat quality				
Marbling score	3.17 ± 2.64^ab^	2.65 ± 1.60^b^	4.17 ± 2.14^ab^	4.71 ± 2.02^a^
Meat color	4.50 ± 0.55^a^	4.96 ± 0.22^a^	4.83 ± 0.75^ab^	4.64 ± 0.50^a^
Fat color	2.67 ± 0.52^b^	3.00 ± 0.00^a^	2.83 ± 0.41^ab^	3.00 ± 0.00^a^
Texture	18.17 ± 4.40^a^	19.60 ± 3.42^a^	16.83 ± 4.58^a^	15.57 ± 4.22^a^
Maturity	2.00 ± 0.00^a^	2.00 ± 0.00^a^	2.00 ± 0.00^a^	2.07 ± 0.27^a^
Quality grade (1^+^:1:2:3)	33:0:33:33	10:25:45:20	33:33:17:17	36:36:28:0

^1)^40% forage in raising stage and 1^st^ fattening period + concentrate without Cr-Met.

^2)^40% forage in raising stage and 1^st^ fattening period + concentrate with Cr-Met.

^3)^60% forage in raising stage and 1^st^ fattening period + concentrate without Cr-Met.

^4)^60% forage in raising stage and 1^st^ fattening period + concentrate with Cr-Met.

^ab^Significant differences occurred at (P < 0.05).

Table 3 shows that the greatest marbling score belonged to the HFCM group and was significantly higher than LFCM group (P < 0.05). The fat color was significantly lower (P < 0.05) in LF group than LFCM and HFCM groups, whereas HF group didn’t show any difference in fat color among the treatment groups (P > 0.05). Meat color, texture and maturity were not affected by the treatment groups (P > 0.05).

### Blood metabolites

Triglyceride, cholesterol, plasma urea nitrogen (PUN), total protein (TP), and creatinine collected in Table 4 were not affected by treatment groups (P > 0.05). The concentration of high density lipoprotein (HDL) was significantly higher for the HFCM group than LF, LFCM and HF groups (P < 0.05). Low density lipoprotein (LDL) content in LF was the highest among all treatment groups, but it showed a meaningful difference compared to HFCM group (P < 0.05). Concentration of glucose in the HFCM group was significantly higher than in the HF group (P < 0.05), but there were no other differences in glucose content between and within low forage and high forage rations. Based on Table 4 we observed the lowest content of albumin in LFCM group and it was significantly lower than LF and HF groups (P < 0.05), but no difference was perceived among LFCM and HFCM groups. The highest significant level of calcium was found in the LF group compared to others (P < 0.05) except HF group. The insulin rate in the blood was significantly higher for the HFCM group than LF, LFCM and HF groups (P < 0.05).

**Table 4:** **Effects of forage level and Cr-Met chelate supplementation diets on blood metabolites obtained from native Korean beef steers (Hanwoo, Bos Taurus)**

**Item**	**Treatment**			
	**LF** ^**1)**^	**LFCM** ^**2)**^	**HF** ^**3)**^	**HFCM** ^**4)**^
Triglycaride, mg/dl	29.25 ± 9.03^a^	24.42 ± 5.98^a^	31.05 ± 7.78^a^	22.33 ± 3.14^a^
Cholesterol, mg/dl	145.00 ± 15.28^a^	191.31 ± 18.38^a^	201.00 ± 4.24^a^	204.63 ± 17.78^a^
HDL-C, mg/dl	92.75 ± 7.41^b^	118.50 ± 22.41^a^	88.50 ± 7.78^b^	128.25 ± 17.40^a^
LDL-C, mg/dl	102.00 ± 15.98^ab^	87.17 ± 18.25^ac^	111.00 ± 14.14^a^	79.00 ± 13.18^c^
Glucose, mg/dl	49.50 ± 8.96^ab^	61.42 ± 18.46^ab^	41.50 ± 4.95^a^	65.30 ± 20.28^c^
BUN, mg/dl	15.20 ± 2.62^a^	14.92 ± 2.74^a^	13.95 ± 1.20^a^	15.33 ± 1.16^a^
T, Protein, g/dl	6.40 ± 0.14^a^	6.25 ± 0.93^a^	6.55 ± 0.07^a^	6.69 ± 0.70^a^
Albumin, g/dl	4.50 ± 0.24^a^	3.80 ± 0.65^b^	4.55 ± .35^a^	4.10 ± 0.46^ab^
Creatinine, mg/dl	1.38 ± 15.28^a^	1.30 ± 0.23^a^	1.50 ± 0.28^a^	1.39 ± 0.24^a^
Calcium, mg/dl	10.35 ± 0.57^a^	8.05 ± 1.95^b^	9.95 ± 0.49^ab^	8.43 ± 1.21^b^
Insulin, μU/ml	6.64 ± 1.09^a^	7.87 ± 2.37^ab^	9.04 ± 0.67^ab^	9.81 ± 1.57^a^

^1)^40% forage in raising stage and 1^st^ fattening period + concentrate without Cr-Met.

^2)^40% forage in raising stage and 1^st^ fattening period + concentrate with Cr-Met.

^3)^60% forage in raising stage and 1^st^ fattening period + concentrate without Cr-Met.

^4)^60% forage in raising stage and 1^st^ fattening period + concentrate with Cr-Met.

^a-c^Significant differences occurred at (P < 0.05).

## Discussion

### Performance in raising and 1^st^ fattening period

Zinn and Plascencia [19] reported no difference among feedlot cattle fed with high (30%) and low (10%) levels of alfalfa hay supplemented with fat in their initial and final weight gain during the growing-finishing period. The final BW was similar among treatment groups and averaged 577.9 ± 6.6 kg when cows were fed with high (50% hay) and low (12% hay) forage level supplemented with wet distillers grain [12,20,21]. Moreover, according to Ohh et al. [2], the positive effect of Cr supplementation can be associated with its obvious influence on the systematic division of energy between adipose and lean tissue. Higher ADG was found at the end of 1^st^ fattening period in steers fed with a high level of forage than steers fed with low forage levels in this experiment; however, the differences were not significant. Therefore, these results are in agreement with other studies [12,19]. Diets containing more than 8% lipid on a DM basis have been reported to decrease DMI and 8% is suggested as a maximum for beef cattle diets [22]. Boadi et al. [13] concluded that animals that were fed low forage grain diets produce more rumen methane than animals fed high forage grain diets supplemented with sunflower seeds. This fact may explain why no difference was observed in the final weight gain in both groups of high and low forage level.

Steers fed the low forage: grain diet consumed more DM than steers fed the high forage: grain diet during the 126-d feeding period [13]. Dry matter intake was similar (P = 0.78) in period I for steers assigned the low forage: grain (8.3 ± 0.6 kg) and high forage: grain (9.4 ± 0.6 kg) treatment groups; however, the rise in DMI for steers assigned the low forage: grain ration was higher in periods 2 (13.0 ± 0.6 kg) and 3 (13.8 ± 0.6 kg) than for the steers fed with high forage: grain ration in periods 2 (9.4 ± 0.5 kg) and 3 (11.0 ± 0.6 kg). In period I (raising period) of this study, the authors found the same result due to the fact that in low level of forage steers could consume more feed because their rumen has more capacity. Over time, steers tended to consume more feed of high forage level than low forage level. During the final period there was no significant overall difference in DMI between treatment groups. Fluctuation between both treatment groups in feed conversion ratio in every period during raising (I period) and 1^st^ fattening (II) might be explained due to the same fluctuation in DMI. But the overall FCR in 60 F was higher than 40 F. Zinn and Plascencia [19], however, reported that the addition of 6% fat (DM basis) to low forage rations (10% forage) did not affect ADG, reduced DMI, and improved feed efficiency by 3.4%, whereas the addition of 6% fat (DM basis) to high forage diets (30% forage) increased ADG, lessened DMI, and enhanced feed efficiency by 17.5%. Similar results were reported by Kegley et al. [23], but Hong et al. [4] stated that 400 ppb of Cr-Met inclusion in high forage diet improved feed efficiency in Korean steers.

### Carcass characteristics and 2^nd^ fattening period

Reports of lessened fat over the 10th rib and turn down yield grades have been informed in lambs supplemented with chromium tripicolinate [24]. However, Chang et al. [25] announced that supplementing high chromium yeast for beef growing-finishing steers had no effect on carcass characteristics. Barajas et al. [26] reported that supplementation of chromium in beef steer diets has no effect on back fat thickness and rib-eye area.

More ruminal starch digestion should increase the organic acids that are later converted to glucose, which is a precursor for marbling. Boadi et al. [13] observed a marbling grade of A or better for 99% of steers however, the proportion of AA carcasses and rib-eye area were higher (P < 0.05) for the low vs. the high forage diet (75 vs. 33% and 85.9 ± 0.9 vs. 79.7 ± 0.9 cm, respectively).

Chromium supplementation has been employed to manipulate the quality of meat due to its biological function on body fat and muscle metabolism [27]. Any discussion of marbling levels must consider genetic influence. The result of organic chromium supplementation on meat quality in farm animals is varied with animal species, form of dietary chromium, and the level of chromium supplementation. However, the result has varied presumably due to other extrinsic factors such as onset and level of supplementation, nutrients and level of basal diet, breed and species [27-29]. Marbling score in HFCM was higher than LFCM while there was no difference between other treatment groups without Cr-Met supplementation, indicating that Cr-Met supplementation may affect marbling score. This significant difference may be due to the positive effect of chromium supplemented into high forage ration. The function of chromium to regulate glucose level in tissue has recently been hypothesized to produce marbled beef via phase-regulated supplementation of dietary chromium [4]. Due to the result of insulin and glucose concentration in the blood, in this study, those metabolites tended to be higher in HFCM group than LFCM group. As glucose is a precursor for marbling score, and given the role of insulin in using more glucose in tissues like meat, the higher marbling score in the HF group ration in comparison to the LF group can be explained. For this purpose, Cr-Met chelate was suggested since the chelate is generally safer than other organic chromium and could be required relatively more easily than others, due to its higher bioavailability. However, a small difference in results was reported with Korean native steer. 400 ppb of Cr-Met inclusion into high forage diet improved feed efficiency in Korean native steers during the growing period. Serum insulin concentration was the highest in chromium supplemented steers [4]. Higher fat color rate in HFCM group compared to LF group may be explained by the addition of Cr-Met in HF and LFCM groups due to the fact that steers fed with high level of forage have more beta-carotene, which can be found in high percentages in forages. Higher fat color values in Cr-Met groups may be explained by the presence of chromium in the diets. Chromium in the diets due to higher bioavailability in the form of Cr-Met chelate may cause higher feed digestion and higher fat color values in the treatment groups.

### Blood metabolites

The decrease in blood cholesterol can be affected by Cr-Met because in both LFCM and HFCM groups a lower amount of cholesterol was observed, but which was not significant [6]. The HDL helps to prevent narrowing of the artery walls by removing the excess cholesterol and transporting it to the liver for excretion. The LDL carries cholesterol for cell building needs, but leaves behind any excess on artery walls and in tissues [4,6]. High LDL and low HDL levels indicate diets high in refined carbohydrates and/or carbohydrate sensitivity. Low levels of HDL are strong indicators of insulin resistance, but in our experiment we observed adverse amounts due to using Cr-Met in diets. Supplemental chromium also plays an important role in serum cholesterol homeostasis. Chromium supplementation decreased the level of the blood’s total cholesterol, LDL cholesterol, and triglyceride but increased the level of HDL cholesterol [30]. Dietary saturated fat and cholesterol lead to elevated levels of cholesterol in the blood.

A possible mechanism of chromium on amino acid synthesis has been predicted due to the role of insulin in amino acid uptake, but other activities of chromium in protein metabolism have not been reported [31]. However, in this study, HDL cholesterol was remarkably increased whereas LDL cholesterol was decreased, resulting in a net increase of total cholesterol. In studies with rats, Kim et al. [32] showed that serum total cholesterol was decreased, whereas HDL cholesterol was increased by 300 ppb of Cr-Met addition when rats were fed a high fat diet. In relation to this result, Kim et al. [32] reported that rats fed Cr-Met chelate at 0, 300, 600, 1,200 ppb of chromium levels exerted a decrease in obesity index. These results showed a dosage-dependent decrease in body fat with increasing the dosage of Cr-Met and suggested intervention by Cr-Met in lipid metabolism. A physiological function of dietary chromium in animals and humans is proposed [7] in the view of carbohydrate metabolism, lipid metabolism, protein synthesis, growth, and longevity. Schwarz and Mertz [33] had recognized chromium as a component of GTF, which enhance not only tissue sensitivity to insulin but also glucose utilization. Therefore, glucose uptake, glucose use for lipogenesis, glucose oxidation to carbon dioxide, and glycogenesis are increased by the addition of chromium plus insulin to animal tissue [34].

## Conclusions

Results of the raising and 1^st^ fattening periods suggest that rumen characteristics and digestive tracts of Korean native steer were developed by the feeding of high forage-based diet during the raising period and showed a good approach for the growing steer which constantly achieved ADG during the 1^st^ fattening period.

The results of the 2^nd^ fattening period indicate that supplemental Cr, as Cr-Met chelate, improved the quality grade of meat and blood metabolites in the beef steer. The effectiveness of Cr-Met supplementation was higher in high-forage feeding than low-forage feeding of diets in the 2^nd^ fattening period.
